# Unsupervised Image-to-Image Translation: A Review

**DOI:** 10.3390/s22218540

**Published:** 2022-11-06

**Authors:** Henri Hoyez, Cédric Schockaert, Jason Rambach, Bruno Mirbach, Didier Stricker

**Affiliations:** 1 Paul Wurth S.A., 1122 Luxembourg, Luxembourg; 2 Department Computer Science, Technische Universität Kaiserslautern, 67663 Kaiserslautern, Germany; 3 German Research Center for Artificial Intelligence (DFKI), 67663 Kaiserslautern, Germany

**Keywords:** unsupervised image-to-image translation, machine learning, computer vision, deep learning, generative adversarial networks, review

## Abstract

Supervised image-to-image translation has been proven to generate realistic images with sharp details and to have good quantitative performance. Such methods are trained on a paired dataset, where an image from the source domain already has a corresponding translated image in the target domain. However, this paired dataset requirement imposes a huge practical constraint, requires domain knowledge or is even impossible to obtain in certain cases. Due to these problems, unsupervised image-to-image translation has been proposed, which does not require domain expertise and can take advantage of a large unlabeled dataset. Although such models perform well, they are hard to train due to the major constraints induced in their loss functions, which make training unstable. Since CycleGAN has been released, numerous methods have been proposed which try to address various problems from different perspectives. In this review, we firstly describe the general image-to-image translation framework and discuss the datasets and metrics involved in the topic. Furthermore, we revise the current state-of-the-art with a classification of existing works. This part is followed by a small quantitative evaluation, for which results were taken from papers.

## 1. Introduction

Image-to-image (I2I) translation aims to transfer an image from one domain to another while preserving the content of the given image. For example, we can take the famous horse to zebra translation, where the aim is to translate a horse image into a zebra image. The domains of zebra and horse images differ by some of characteristics given by the two sets of images (for example, zebras have stripes, and horses have bigger tails than zebras.)

**Figure 1:**
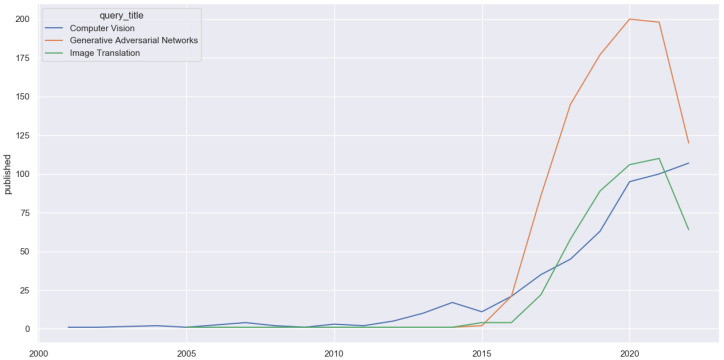
Number of papers published per year in deep learning, generative adversarial networks, image-translation and image-to-image translation. Papers from the ArXiV database were counted.

As shown in Figure 1, the number of publications in image translation increased when generative adversarial networks (GANs) were proposed. In 2016 Isola et al. presented Pix2Pix [1], a conditional model that is able to translate an image from one domain to another using paired training. This work was followed by Pix2PixHD [2], which was translation of high-resolution images. However, even though these methods performed well when they were significant advances over the state-of-the-art, one major problem of these paired image-to-image translation methods is the paired dataset, a dataset where an image already has its translated counterpart. These paired datasets are hard, expensive or even impossible to obtain due to the parity constraint between the input and output.

Consequently, the research community has explored approaches to overcoming the need for paired datasets. For example, Bousmalis et al. [3] tried an unsupervised image-to-image translation method with a domain adaptation on the pixel space. With a more probabilistic point of view, Liu et al. [4] proposed an image translation method based on the shared latent space assumption. In the same way, Taigman et al. [5] proposed another architecture composed of a GAN and an input function *f*. The GAN is trained to generate wanted images and *f* converts an image to a latent representation, which is used by the GAN for the generation.

In 2017, Zhu et al. introduced CycleGAN [6], a network able to translate an image from one domain to another using cycle consistency. Nevertheless, despite this method breaking the paired constraint, it requires a lot of training iterations and suffers from instability. Since then, many applications have emerged (e.g., semantic to real [1,6,7], maps to satellites [1,8] and satellites to street view [9]).

There remain challenges, the most prominent being:

**Complex Translations:** Translations that need heavy modifications to match the target space, such as geometrical transformations; or diverse images, such as those of landscapes.

**Effective Training:** In the context of unsupervised translation, cycle consistency is often used. However, it can be too restrictive in some cases, such as glass removal tasks [10]. This case forces the model to hide information in the translation in order to allow the backward translation to satisfy the cycle consistency [11]. Since that time, some implementations try to do without cycle consistency by proposing alternative losses. On the other hand, other approaches try to propose new discriminator architectures to stabilize the training.

**Data Scarcity:** Sometimes data are missing or are particularly difficult to collect. Some works have tried to reduce the data requirements of translation models by augmentation methods or directly with architecture changes.

This paper is aims to give an overview of principal works on unsupervised image-to-image translation (UI2IT), along with the current challenges and limitations. To accomplish these ends, this review is organized as follows: In the first place, we detail the general process of UI2IT. Secondly, we give an overview of datasets and metrics used for UI2IT, followed by the classification of methods. Thereafter, the main parts of this review are presented, namely, architecture changes, complex translation, data issues, attribute editing, guidance, disentanglement learning and contrastive learning methods. A short discussion about method comparison and current challenges is presented afterward, followed by the conclusions of the article.

## 2. Problem Description

Image-to-image translation aims to convert an image $x_{A}$ from a source domain $D_{A}$ into an image $x_{B}$ in a target domain $D_{B}$ while preserving the content of $x_{A}$, and vice versa. More formally, it consists of two functions, *F* and *G*:

(1) $$\begin{array}{c} G_{\theta }:D_{A}\to D_{B}\\ F_{\Phi }:D_{B}\to D_{A} \end{array}$$

where θ and Φ are learnable parameters. For example, in the horse to zebra translation task, *F* translates a horse photo to a zebra photo, and *G* does the reverse. From the deep learning point of view, *F* and *G* are generators and are trained against two models called discriminators, which aim to discriminate real images from generated images. As generators and discriminators are competing against each other, this training technique is defined as “adversarial training”.

In the early stage of I2I translation, the translation task consists of texture transformations, such as horse ↔ zebra, or season transfer. Nowadays, with the evolution of translation methods, the “content” and “style” significance have changed. The “content” features are more related to specificities we want to preserve during the translation, as opposed to “style”, which defines features we need to change.

### Losses

Since we know which models are involved in I2I translation, we will briefly go over the learning objective of unsupervised image-to-image translation. We define $G_{A\to B}$ as the generator, which translates an image from the domain $D_{A}$ to $D_{B}$ and vice versa. We also define $D_{A}$ and $D_{B}$ as the discriminators.

**Adversarial Loss:** The adversarial loss [12] $L_{GAN}$ symbolizes the adversarial interaction between generators and discriminators. For example, $G_{A\to B}$ tries to make a translation of $x_{A}$ that resembles an image in the $D_{B}$ domain and $D_{B}$ tries to make the distinction between real samples $x_{B}$ and translated samples ${\tilde{x}}_{B}=G_{A\to B}\left(x_{A}\right)$.

**Cycle Consistency Loss:** The cycle consistency loss was introduced by Zhu et al. in the CycleGAN paper [6]. After describing that a given translation from X→Y may not preserve all the characteristics of an image in *X*, since there is an infinite number of mappings between X→Y, the authors claimed that translation should be cycle-consistent. Therefore, for an image $x_{A}$ from $D_{A}$: $x_{A}\to G\left(x_{A}\right)\to F\left(G\left(x_{A}\right)\right)\approx x_{A}$ and vice versa. This loss facilitated the use of an unpaired dataset but encouraged the generators to pass unnecessary information during the translation, thereby degrading performances [11].

**Identity Loss:** To further encourage the mapping that conserves the source image features, Taigman et al. [5] introduced a regularization term to restrict generator translations. This loss minimizes $\left|\right|G_{A\to B}\left(x_{A}\right)-x_{A}{\left|\right|}_{1}$, forcing the generator to translate relevant features (e.g., shapes and borders).

**Full Objective:** Finally, the full objective function can be formulated as the weighted sum of all previously listed losses, where $\lambda_{GAN}$, $\lambda_{cyc}$ and $\lambda_{id}$ denote parameters which control the relative importance levels of the adversarial loss, the cycle consistency loss and the identity loss, respectively. When these models are correctly trained and stabilized, the generator produces good results and the discriminator’s predictions are nearly random.

## 3. Datasets and Metrics

**Figure 2:**
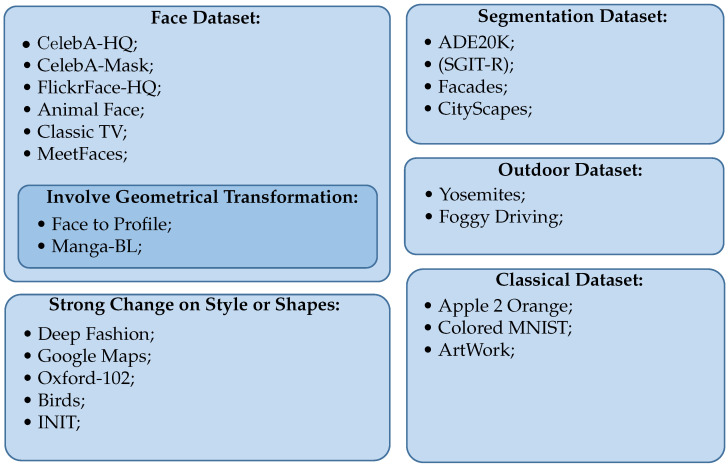
Dataset organization where we classify datasets given the main characteristics.

Since we gave a general introduction to image-to-image translation, in this part, we describe not only the most popular datasets but also datasets introduced by papers of our literature review. Furthermore, Figure 2 shows these datasets sorted by category, which depends on the translation task or highlighted challenges.

### 3.1. Datasets

#### 3.1.1. Face Datasets

- **(2017) Animal Face High Quality (AFHQ):** AFHQ contains animal faces in 512×512 resolution and contains images of dogs, cats and wild animals. It was introduced by Choi et al. [13].
- **(2018) CelebA-HQ:** CelebA-HQ is the high-quality version of the CelebA dataset. It was introduced by Karras et al. [14] and contains 30 K images of 1024×1024 pixel face images.
- **(2018) Flickr Face High-Quality (FFHQ):** FFHQ contains 70 K high-quality human faces and can be seen as an upgraded version of the CelebA dataset, as it stores more variation in terms of ethnicity and image background. It was introduced in the StyleGAN paper by Karras et al. [15].
- **(2019) CelebAMask-HQ:** It was introduced by Lee et al. [16]. This dataset contains 30 K CelebA-HQ images and fine-grained semantic annotation.
- **(2020) MangaGAN-BL:** MangaGAN-BL is a dataset introduced by Su et al. [17]. It was derived from a manga work called Bleach and contains facial features such as facial landmarks and bodies. It can be used on translation tasks with big gaps between the source and the target domains.
- **(2020) MeetFace:** MeetFace is another face dataset proposed by Karras et al. [18] and contains 1336 high-quality face images.
- **(2021) ClassicTV:** It is a dataset published by Nederhood et al. [19] containing face crops from a 1960s TV show (i.e., Bonanza and the Lucy Show).
- **(2022) Front → Profile Face Dataset:** Introduced by Xu et al. [20], the task is to translate a given front face image into a profile face. This task involves geometric transformations and needs a strong translation ability.

#### 3.1.2. Large Changes in Style or Shape

- **(2008) Oxford-102:** Alternatively known as the Flowers Dataset, this dataset contains images of 102 flower categories introduced by Nilsback et al. [21]. This dataset was used by Kim et al. [22]; they translated a flower from one specie to another.
- **(2016) DeepFashion:** This dataset was introduced by Liu et al. [23] as a dataset with 46 categories and 1000 descriptive attributes. This dataset contains photographs from shop presentations and consumer photos.
- **(2019) INIT Dataset:** This dataset contains a set of images taken in Tokyo, Japan, with a camera adapted for autonomous driving experiments (i.e., SEKONIX AR0231). It contains four classes (e.g., Sunny, Night, Rainy and Cloudy) and was introduced by Shen et al. [24].
- **(2020) Bird Dataset:** Taking the same idea as AFHQ, this dataset was introduced by Van Horn et al. [25] and contains 48,562 images of 55 bird categories. It can be useful for image translation through labels [26].
- **(2022) Google Map:** This dataset was introduced by Song et al. [27] for MapGenGAN and consists of real-looking map images translated from Google satellite-view images of New York City. This dataset needs a precise translation model to correctly translate aerial road views into Google Maps roads. However, since this dataset can be scrapped for other cities [28], comparing models based on this paper’s results is not feasible.

#### 3.1.3. Segmentation Dataset

- **(2016) Cityscape:** CityScapes is a widely used dataset used for the semantic segmentation of urban scenes. This dataset contains nearly 5000 fine-grained images and 20K coarse annotations. This dataset was recorded in several cities during several months and time conditions. With this dataset, Cordts et al. [29] provided a strong dataset for urban environment segmentation.
- **(2017) ADE20K:** Following the segmentation trend, Zhou et al. [30] also published a versatile, fully annotated dataset. This dataset contains nearly 20K images for training with fined-grained, annotated images with 150 semantic categories.
- **(2017) Facade Dataset:** This is another dataset used to check the semantic translation of models. It was introduced by Tylecek et al. [31].
- **(2021) SGIT-S, SGIT-R and SGIT-C:** This dataset answers the needs of saliency-guided image translation (SGIT) and was proposed by Jiang et al. [32]. SGIT-S contains synthetic images generated from the CLEVR project [33], which contains a set of images containing objects with complex backgrounds. SGIT-R is a saliency-guided real-world image dataset, for which saliency maps were produced by a mouse contingent experiment conducted on seven participants. Finally, SGIT-C can be seen as the evaluation set of randomly sampled images of Place2 [34] and photos in difficult settings.

#### 3.1.4. Outdoor Dataset

- **(2017) Yosemite:** This dataset was also published in the CycleGAN paper [6]. It contains photographs of Yosemite park taken in summer and winter for the season transfer task. The photographs were downloaded from Flickr.
- **(2017) Foggy Driving:** Introduced by Sakaridis et al. [35], this synthetic dataset was derived from CityScapes and was made by adding fog into real, sunny images.

#### 3.1.5. Classical Dataset

- **(2017) Apple ↔ Orange:** This dataset was also released in the Zhu et al. paper [6]. Similarly to horse ↔ zebra, these samples came from ImageNet [36] and were scaled up to 512×512.
- **(2017) Colored MNIST:** This dataset is an upgrade of MNIST proposed by Gonzalez-Garcia et al. [37]. Variations were introduced to the original MNIST dataset, such as background modifications and changes in the number of colors.
- **(2017) Artwork:** This dataset was created by Zhu et al. [6]. It was constructed by downloading photographs from Wikiart and Flickr and contains paintings from Van Gogh, Monet, Cezanne and Ukio-e.

### 3.2. Metrics

To evaluate an I2I translation model, various metrics have been defined. In the beginning, human perception is used to give a score on generation quality. However, human perception is not a time-consistent metric, since an individual’s perception can vary over time. This was reflected by the introduction of new evaluation metrics. In supervised I2I translation, since a given translation can be directly compared to the ground truth, simple evaluation metrics such as mean-squared error (MSE) or the perceptual distance (PD) [38] can assess model performance. However, UI2IT cannot take advantage of this; hence, researchers tend to use different evaluation metrics to compare UI2IT methods.

The most popular metric is Fréchet inception distance [39] (FID), usually followed by learned perceptual image patch similarity [40] (LPIPS). The former evaluates the generation quality and the latter use features of a pretrained model to estimate the diversity of generations. LPIPS is also known to be correlated with human perception.

**Figure 3:**
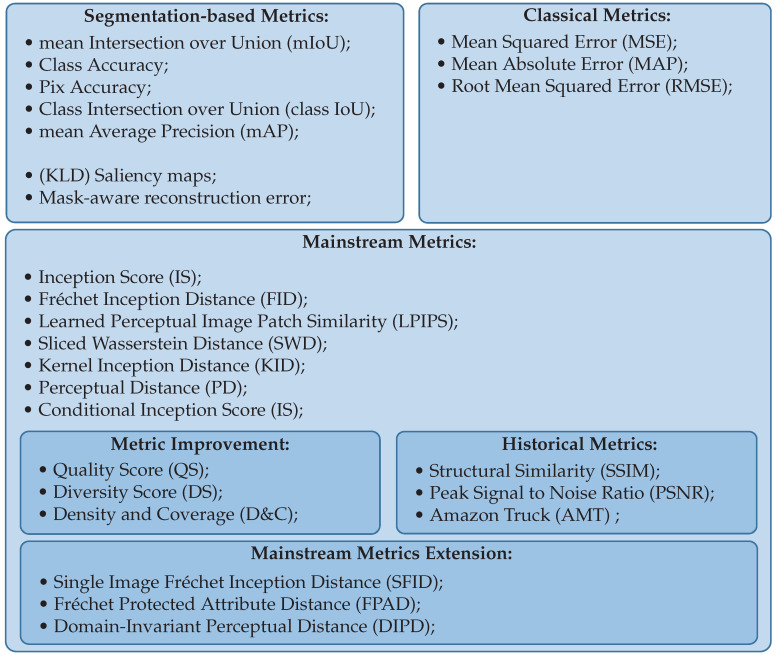
Categorization of metrics their main characteristics.

Since the release of FID in 2017 and LPIPS in 2018, researchers had to face new challenges, and new metrics have been released to perform a better comparison or face specific challenges. In this part, we list and explain well-known metrics and also introduce the latest metrics. We also provide Figure 3, which aims to guide the reader through the most suitable metrics.

- **(2004) Structural similarity (SSIM):** Structural similarity (SSIM) [41] was developed as an image compression metric. The idea of this metric was to estimate the similarity in terms of the structure between two images. The higher, the better.
- **(2005) Peak signal to noise ratio (PSNR):** Another image compression metric is peak signal to noise ratio (PSNR) [42], which can assess the quality of a compression algorithm. The higher, the better.
- **(2005) Amazon Mechanical Truck (AMT):** AMT is a micro-work platform that allows human perception evaluation. In UI2IT evaluation, users are usually asked for diversity or realism notations.
- **(2016) Perceptual distance (PD):** Johnson et al. [38] found that pixel-based loss is sensible to little perturbations which do not change the content of an image too much, such as pixel shift. Inspired by works which use perceptual loss functions, they proposed to train a Feed-forward Transformation Network to minimize a perceptual loss function and hence provide features that are more robust to pixel perturbations. The lower, the better.
- **(2016) Inception score (IS):** The inception score (IS) is a metric created by Salimans et al. [43]. They aimed to remove the dependency on human evaluation. Hence, this metric evaluates the generation quality of GANs using predictions of the well-known Inception v3 classification model introduced by Szegedy et al. [44] to describe the quality of the classification coincidence and the variety in the distribution of these classifications. The closer to 1, the better.
- **(2017) Fréchet inception distance (FID):** FID is the most used metric in I2I translation. This metric has been introduced by Heusel et al. [39] as an improvement of the inception score (IS). They claimed that IS does not take into account the real and synthetic statistics in the score. On the contrary, FID estimates the quality of a given generation using Inception v3’s [44] features from real and generated images. These features are then used to calculate the Earth mover’s distance, also known as the 2-Wasserstein distance. The lower, the better.
- **(2018) Sliced Wasserstein distance (SWD):** Estimating the generation of models often corresponds to estimating the distance between two probability distributions. To estimate this distance, the Wasserstein distance gives good theoretical guarantees. However, it takes a long time to calculate, especially with large-scale problems. Hence, sliced Wasserstein distance (SWD) has been proposed by Karras et al. [14] as a more computationally efficient distance measure. The lower, the better.
- **(2018) Learned perceptual image patch similarity (LPIPS):** [40] As a metric correlated with human perception, the LPIPS metric is proposed as a good solution to calculate the diversity of image translation models. Like FID, it does not exploit low-level pixel relations but does have features from a pretrained AlexNet. The higher, the better.
- **(2018) Domain-invariant perceptual distance (DIPD):** The DIPD is a modification of the perceptual distance which does not work well in the unsupervised setting, since it needs an input image and a reference image. However, this perceptual distance remains useful for content preservation tests. To make this metric more domain-invariant, Huang et al. [45] proposed to perform an instance normalization of the VGG output features to erase the most domain-specific features. The lower, the better.
- **(2018) Conditional inception score (CIS):** The CIS is a modification of the inception score (IS) proposed by Huang et al. [45]. Instead of IS, it computes the IS conditioned on one input image, encouraging models to not be deterministic. The higher, the better.
- **(2018) Kernel inception distance (KID):** [46] The Kernel inception distance (KID) is a variation of FID. The KID is similar to FID, since it also uses inception scores but is also different because it calculates the square maximum mean discrepancy (MMD) of the inception features. The lower, the better.
- **(2019) Single-image Fréchet inception distance (SIFID):** For those who are more interested in the internal statistics of an image, Shaham et al. [47] proposed SIFID. The specificity here is, instead of using the features of the last pooling layer, the authors use the features of the previous layer for the computation of the FID. The lower, the better.
- **(2020) Density and coverage (D&C):** This metric introduced by Naeem et al. [48] is known to be more robust to outliers and can be tested on more uncovered domains of ImageNet. It estimates the diversity and the fidelity of the model output with a KNN-based method. The higher, the better.
- **(2020) Quality score (QS) and diversity score (DS):** The quality score (QS) and diversity score (DS) were introduced by Gu et al. [49]. This metric aims to evaluate the quality and the diversity of each sample of a translation model. The key here is that these metrics evaluate the quality and the diversity separately. The higher, the better.
- **(2020) Fréchet protected attribute distance (FPAD):** One of the latest metrics is the Fréchet protected attribute distance (FPAD), another modified version of FID. For this metric, Hwang et al. [50] proposed to extract protected features using their proposed protected attribute classifier (PAC) and then compute the FID between the generated and real protected features. The lower, the better.
- **Intersection over union (IoU):** Intersection over union is a standard metric in object detection for image segmentation. It corresponds to the overlap between two areas divided by the total area occupied by these two boxes. In I2I translation, these segmentation metrics are also useful to evaluate translation performances. The nearer to 1, the better.
- **Mean average precision (mAP):** In classification models, precision and recall are useful metrics for evaluating a given model. However, in object detection methods, the prediction is also determined by a threshold which can influence the precision and recall. This metric is introduced as a less arbitrary metric. The higher, the better.

## 4. Classification of Image Translation Method

As this literature review is meant to give the reader a comprehensive understanding of the actual I2I translation field, we classified papers based on their main contributions. The categories follow:

**Architecture Change:** Due to the aforementioned challenges and new module propositions, some works exploit contributions coming from other fields [51,52]. Nevertheless, some proposals made changes specific to I2I translation [53].

**Complex Translation:** In recent years, there has been a research interest in particularly difficult translations. This category introduces recent works that aim to bridge between highly different domains.

**Data Issues:** Generation models usually need a lot of data. However, in some cases, the data cannot be available in sufficient quantity. This part summarizes recent attempts to alleviate the data dependency.

**Attribute Editing:** Instead of translating the entire image, some works pay more attention to local feature modifications. In this part, we talk about works that force the model to translate specific features.

**Guidance:** This part talks about the different ways to guide the translation models.

**Disentanglement Learning:** Methods are based on the assumption that an image can be divided into specific sub-spaces. These sub-spaces can be used in order to translate an image with a particular content to a given style.

**Contrastive learning methods:** Contrastive learning is a self-supervised learning approach. In I2I translation, these methods try to solve inherent problems of cycle consistency. This subpart will describe general contrastive learning methods and summarize the current state-of-the-art in this sub-field.

## 5. Architecture Changes

Since the release of CycleGAN in 2017, some architectural changes have been released to alleviate some drawbacks of previous UI2IT methods. For example, Choi et al. [13] pointed out the deterministic behavior of CycleGAN. They hence proposed an architecture called StarGAN composed of two elements, the former being a generator that translates an image conditioned by the target domain vector, and the latter a multi-task discriminator. This architecture has further been improved with StarGANv2 [54], which introduces a new paradigm with three networks: a style encoder, a generator, and a multi-task discriminator.

Nowadays, in the era where smartphones are among the most used devices, researchers aim to develop faster, lightweight models, as in the case of the work of Chen et al. [55], who used the generator’s encoder as a part of the discriminator. To stabilize the training process, they trained this common part with the discriminator objective. Following the idea of making less heavy models, Shaham et al. [53] proposed a lightweight model that can be trained faster called ASAP-NET, which is composed of a small convolutional encoder and a spatially-varying pixelwise MLP. With this architecture, they qwew able to generate good translation within 35ms for an image of 1024×1024 pixels on an Nvidia GeForce 2080ti GPU. Following the idea to train fewer models, Richardson et al. [56] trained an encoder for the W+ space of StyleGAN [15] using a pyramidal feature extractor.

In general, a good translation model is a model that can generate diverse images. In medicine though, translation models should generate precise translations and produce unique images for each patient. Shen et al. [57], inspired by the work [24,58], proposed a model able to perform a unique translation by forcing the model to be self-inverse (e.g., $G=G^{-1}$).

Currently, some research projects address high-resolution translation. The higher the quality of the images, the more complicated the translation task. To be able to perform these translations, Shao et al. proposed SpatchGAN [59] to improve PatchGAN by releasing an architecture that is more able to shape changes and hence stabilize the training on challenging translation tasks. This model takes an image at different scales to be sure that this discriminator captures both global and local information. Another work that aims to generate high-resolution images is LPTN from Liang et al. [60]. They profited from Laplacian pyramids to make high-resolution translations.

In another work, Gao et al. [10] stated that cycle consistency forces the model to hide information to guide the model for the reverse translation and satisfy the cyclic constraint. To alleviate this issue, they proposed a wavelet-based skip connection to filter out low-frequency components inside of image information and propagate the high-frequency information directly at the end of the generator. Furthermore, they proposed a discriminator to force the model to generate highly detailed generations and a new loss called attribute regression loss.

## 6. Complex Translations

Some methods manage to generate visually pleasing images. StarGAN manages to transfer images of faces while keeping them realistic. However, face translations are still less challenging [61], since the faces are centered. Nowadays, articles also focus on more difficult translations, requiring geometrical transformations or a big gap between the domains (face to anime; image deraining).

### 6.1. Large Domain Gaps

Recently, the research workforce has focused on challenging translations either by architecture proposal or dataset. For example, Kim et al. [61] proposed a dataset called selfie-to-anime which introduces another translation challenge, as the shape of real faces has to be modified in order to get good translation results. To reinforce the spatial transformation capabilities of neural networks, Jaderberg et al. [62] introduced a spatial transformer module that can be used in any neural network. Another work from Fu et al. [63] introduced a geometry-consistent paradigm, the main idea being that the semantic information of a given image should not be affected by simple transformation and used this idea in one-sided image translation. This idea is also used in [7].

Li et al. [64] noted that niche problems do not have a lot of data, as is the case for laboratory animals. They proposed a synthetic-to-real approach to alleviate the pose detection model’s performances with a hierarchical translation method.

Zhao et al. [11] proposed to work on the cycle consistency loss directly. They propose ACL-GAN, which uses a proposed adversarial cycle consistency loss to prohibit the model to hide artifacts during the reverse translation.

In the same vein, Yanwu et al. [20] introduced a new method that uses a perturbator *T* and a generator *G*. This method is called maximum spatial perturbation consistency (MSPC). In this method, *T* aims to maximize the distance between G(T(X)) and T(G(X)) and *G* tries to minimize it. At the end of the training, *G* and *T* should be commutative. They stated that their method is a generalization of the geometric consistency, and they also introduced a challenging translation task called front → profile, along with an accompanying dataset.

Following this idea, Zhou et al. [65] introduced Rotate-and-Render to alleviate the problem of front-face photo synthesis from a profile face image. Their paper introduced an unsupervised profile for front synthesis using a 3D-fitting network and a neural renderer.

### 6.2. Progressive Learning

Recently, coarse-to-fine generation models such as diffusion models have contributed to the image synthesis problem. They are able to outperform traditional GAN techniques in image synthesis [66]. However, these methods can suffer from long inference times due to the iterative process of the generation. Due to these achievements, some recent works present translation methods that exploit the power of the coarse-to-fine methods.

Jiang et al. [67] introduced a versatile method for diverse I2I translation tasks which has two streams, one to learn the spatial features and another to learn the style features, and it mainly works on normalization layers.

Lin et al. [68] introduced a coarse-to-fine approach that works with two unpaired images. To achieve this, they proposed TUIGAN to progressively transfer an image from one domain to another in a coarse-to-fine framework. Specifically, they used two pyramids of generator and discriminator to transfer global and local features progressively.

With the idea of translating progressively, Zhou et al. [69] proposed a new version of CoCosNet [70] by making it able to generate high-definition images using a GRU-assisted progressive translation. From another point of view, Ren et al. [9] stated that translation models are able to transfer images that shares some similarities. They stated that one reason for this inability to bridge big domain gaps is the one-pass translation instead of doing the translation progressively. They hence proposed CrossMLP-Mixer to make the translation progressive. They performed this translation on a satellite to street-view task which involves strong geometric transformations.

From another point of view, despite the inference time inherent to the architecture, some researchers investigated diffusion-based I2I translation methods.

**Figure 4:**
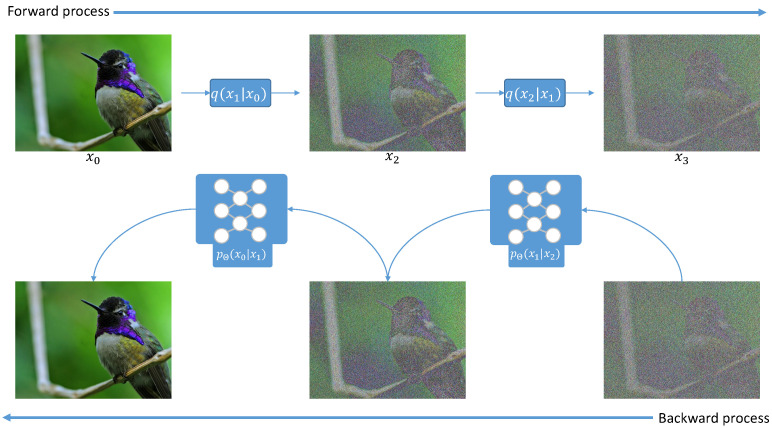
The diffusion process. Image taken from the Creative Commons website and has been released to the public domain by the author.

Diffusion-based architectures are able to generate realistic images of high quality. It has been shown that diffusion models outperform GANs [66,71] in many cases. As shown in Figure 4, denoising diffusion probabilistic models (DDPM) are defined by the task of progressive denoising. In particular, it is defined in two distinct parts, e.g., the forward diffusion and the reverse diffusion. The former is defined by the progressive addition of noise in an image (more precisely a Markovian diffusion process), and the latter is a denoising function that we want to learn.

Thereafter, this method has been widely applied and improved for image synthesis and audio synthesis [72,73,74]. However, one major drawback is the generation speed because of the Markovian process. The given DDPM was 10 to 50 times slower than the GAN counterpart. This problem was recently solved by Song et al. [75], who proposed to transform the Markovian process to a non-Markovian one, and hence proposed denoising diffusion implicit models.

Saharia et al. [71] found that diffusion-based models were not utilized in image-to-image translation and proposed palette, an image-to-image diffusion model able to achieve various tasks, such as image inpainting, uncropping JPEG restoration and colorization, without architecture or hyperparameter changes. This work was shortly followed by UNIT-DDPM from Sasaki et al. [76], the first method that brings a diffusion-based method to the UI2IT paradigm without using adversarial constraints and by using a dual-domain Markov chain generative model. It gives interesting results when compared to mainstream models as CycleGAN or others.

### 6.3. Image to Map Translation

Converting a satellite image to a suitable map involves paying attention to the geometry and converting a colorful image into a minimalist style.

Song et al. [27] adds another argument by saying that making a map is time-consuming and requires a lot of human effort. To alleviate this problem, they proposed MapGenGAN. However, despite the cycle consistency being used in a wide range of applications, this cycle consistency hardly takes into account spatial and geometric features which do not meet the map generation constraints. They also proposed to change some layers of UNET to integrate “Basic Residual Blocks (BRB)”.

Shortly thereafter, Song et al. [28] introduced another satellite-to-map model. They state that the previous satellite-to-map translation methods cannot generalize to diverse city structures. In order to address this problem, they propose remote sensing maps translation (RSMT) as a more general model powered by attention mechanism and guided by a proposed “maps consistency loss”.

### 6.4. Image Deraining

Image deraining can also be considered as a challenging I2I translation task, since getting a fully derained and clean image requires some geometric transformations, such as removing water drops on a windshield. This interest is not new, since Garg et al. [77] published an article in 2004, where they developed a rain removal model based on rain’s physical properties. Data-driven methods followed in 2017, when Fu et al. [78] released a deep learning method based on a deep residual network (ResNet). However, this model was trained on paired simulated data.

Pizzati et al. [79] stated that state-of-the-art models are only able to change the global style with changes in illumination and colors when the gap between domains increases. Therefore, they proposed to help the model to better transform a given rainy image with simple sub-domain images automatically learned from web-crawled data.

Another line of work came from Ye et al. [80], who deplored that deraining models are trained on simulated images that are not realistic enough to be able to correctly learn this complex task. They also stated that a good rainy-image simulation would help the deraining model to be more accurate. Hence, they proposed to learn both at the same time, meaning that they learn to simulate good rainy images and clear images in a disentanglement setting.

## 7. Data Issues

Translation networks are mostly generative networks. Generative networks need a large amount of data to be trained. However, in the world of data, data can be skewed or present in small quantities. This lack of data can create some problems described in the article by Karras et al. [18]. This section explicitly attempts to summarize strategies to reduce the amount of data needed for training.

### 7.1. Few-Shot Learning

In classical deep learning problems, a lot of data have to be used to accomplish a given task. However, in some cases, data are not available in reasonable quantity, and hence the model can overfit. Due to that, researchers try to make methods that do not have to be trained on big datasets. The first method attempted was by Fink et al. in 2004 [81]. They proposed a two-stage method. The first stage is to learn a distance metric where the distances between similar objects are small and the distances between objects of different instances are large. Then, by training the nearest neighbor classifier, it classifies correctly given the knowledge acquired by the metric. Shortly after, Fei-Fei et al. [82] proposed to—instead of learning from scratch—exploit knowledge previously. They tested this idea on a Bayesian approach.

The I2I translation task on limited data was really introduced in 2019 when Liu et al. [83] supposed that humans’ generalization capability comes from past experiences. They hence introduced the first unsupervised few-shot image-to-image translation (FUNIT). To simulate the past experiences, they trained their translation models on a dataset containing diverse images. Their method leverages image translation using few samples based on a generative adversarial network and an architecture modification.

This work was followed by Saito et al. [84], who stated that leaking happens between the content and the style due to the lack of supervision controlling precisely the transferred features. They call this the content loss problem. To solve this problem, they proposed COCO-FUNIT as an improvement of FUNIT. They proposed a modification of the style encoder of FUNIT to prevent information leakage.

Shortly after, Lin et al. [68] performed one-shot unsupervised image translation using only one image in each domain in a progressive learning framework.

### 7.2. Limited Data

Training GANs is known to be data-consuming, needing as much information as possible to approximate the data distribution. However, real-world datasets are not perfectly made. To this end, some works are focusing on the limited data setting.

For example, Cao et al. [85] stated that traditional augmentation techniques can be employed to improve the discriminator’s performance but cannot bed the generator. Facing this problem, they presented ReMix, an augmentation method in the latent space, and they also proposed a new loss called perceptual content loss.

Additional work by Wang et al. [26] proposed a self-supervised initialization scheme to initialize the generator on a given task without any data. They also proposed “source target initialization”, which takes a pretrained model (StarGAN) on the source target data. As less data means that deep layers cannot be correctly learned, they proposed an auxiliary generator to guide deep layers during training.

Later on, Park et al. [86] introduced CUT, who stated that cycle consistency is often too restrictive. Hence, they introduced a contrastive learning method that enables one-sided translation with a maximization of the mutual information between patches. They also claimed that their method reduces data usage and training time.

With the idea of reducing data usage, Baek et al. [87] proposed a truly unsupervised image translation (TUNIT) method which can also work on limited data and can even beat FUNIT. They proposed a guiding network that acts as an unsupervised classifier and a style encoder. However, this method needs some labeled data to efficiently work under a limited data scheme.

### 7.3. Sampling Issues

Another problem in real-world data is unbalanced datasets where the frequency of a given label is higher than those of others. In the UI2IT setting, the model can focus on easy (e.g., modeled by the majority of the samples) cases and discard the harder ones (e.g., rare cases).

The paper of Cao et al. [88] tried to make model training more efficient. It states that the majority of samples are “easy” for the translation models, since they are present in a sufficient quantity. Hence, they proposed to modify the training loop to oversample rare images so the model can take advantage of the most difficult sample to learn.

On the other hand, Xie et al. [89] claimed that datasets can be unaligned. For example, in a face dataset, images taken from a too-different point of view (i.e., medium close-up instead of the usual close-up) can disturb the learning of the model. To get this problem solved, they also proposed dynamic filtering in the training loop to isolate the main data distributions.

As mentioned earlier, UI2IT methods can produce astonishing results [7,60]. However, as these models have a lot of parameters and need a lot of training iterations and data, they are slow to train. Therefore, in their paper, Shaham et al. [53] proposed a much smaller architecture to speed up the training time by making a smaller but smarter model called ASAP-net. The proposed method is composed of a small convolutional encoder and a spatially varying pixelwise MLP.

## 8. Attribute Editing

**Figure 5:**
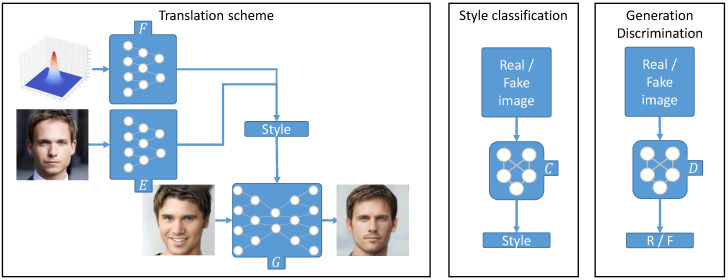
General attribute editing process in I2I translation methods. **E** is the style encoder, **F** is the mapping network that permits the model to explore more modes, **G** is the generator, **D** is the discriminator and **C** is the attribute classifier introduced in [10]. Face images were taken from StarGAN v2 [54].

CycleGAN was a breakthrough in the domain of unsupervised image-to-image translation, as it allows translating from one domain to another in an unsupervised manner. Nevertheless, it does not give a lot of control to the user. Since then, some work emerged with the idea of performing translations given some user constraints. One seminal work was StarGAN by Choi et al. [13], which performs the translation given a source image and predefined labels. However, these predefined labels do not give to the users complete control over the translations. Later, Choi et al. came up with another version of StarGAN called StarGAN v2 [54]. This method introduces another paradigm in UI2IT with three subnets, namely: a generator, a multi-task discriminator and a style encoder. Figure 5 gives a brief overview of the starGAN framework.

Even after the StarGANv2 proposal, researchers tried to add even more control on the generation. For example, Dural et al. proposed FacialGAN [90], which performs translation given a source image, a reference image and a segmentation mask. This method was inspired by StarGANv2, but with a modified version of StarGANv2’s generator, and they proposed a segmentation network to ensure the consistency of the translations. Even though some models can give control to users using a semantic segmentation mask, Liu et al. [91] stated that the segmentation mask does not give complete control over the generations, since the user cannot generate images with clear delimitation on the same face. Hence, they proposed self-adaptative region translation (SART) and introduced region matching loss (RML) and region instance normalization (RIN) blocks. This method ensures that each region is translated separately.

On another hand, Wei et al. [92] stated that a proposed model, such as STGAN [93] makes unnecessary modifications. They also stated that face editing in high resolution is not widely explored. To tackle this problem, they propose Mask-Guided GAN (MagGAN), which is inspired by STGAN. They also proposed a soft segmentation mask and mask-guided reconstruction loss. In addition, they used a multi-level discriminator to stabilize the model’s training. Gao et al. [10] tackled a problem related to cycle consistency. As another work in this review [11], they stated that cycle consistency forces the generator to hide information during the translation to give enough information to satisfy the cycle consistency constraint. To be sure that the generator does not pass any information to satisfy the cycle consistency, they introduced another module called wavelet-based skip connection to ensure that the model does not take the easiest path. The work of Huang et al. [94] talked about reference-based translation and label-based translation. They proposed to combine the best of both worlds by introducing a reference encoding module and a label encoding module.

## 9. Guidance

Although the models can generate translations that can be visually pleasing, training generative neural networks can be complicated because the training is unstable due to the adversarial mechanics involved. This section discusses articles introducing methods to guide models more easily to their final goals. These methods usually use contrastive learning settings first introduced in I2I translation by CUT [86], the authors of which utilized a patch-based method for UI2IT. As mentioned in Section 8, Wei et al. [92] proposed a mask-guided reconstruction loss. This loss ensures that the model is guided to translate only the wanted facial part and hence make high-resolution face editing. To gain a better focus on certain objects during the translation, Bhattacharjee et al. [95] propose to use an object detector to localize objects in a given scene and hence pay more attention to them during the translation. However, the use of this method is computationally inefficient and limited to the objects that the detection model has seen during training. From another point of view, Jeong et al. [96] stated that cycle consistency imposes a determinism between the source and the target domain. They also stipulated that methods apply a global style during the translation, which is problematic for images with multiple objects. They hence introduced a class-aware memory network. This network memorizes the object style to guide the model to the correct translation. In the same vein, Tang et al. also stated that the translation often transfers unnecessary elements of the image. They hence proposed AttentionGAN [51], which uses an attention mechanism to separate the main subject to be translated from the background. Another work from Jiang et al. [52] states that some methods that utilize saliency maps to guide the translation are limited by low-level pixel modifications. They proposed another method that uses the saliency map to guide their translations. They proposed a saliency-guided attention module and saliency-guided global and local discriminators. Another particular work from Pizzati et al. [97] notes that continuous translation requires supervision to realize the intermediate translation. To be able to perform continuous translations in an unsupervised manner, they introduced CoMoGAN. This method introduced a functional normalization Layer which is placed between the encoder and the decoder. This method enables cyclic translation of a particular image for continuous day-night translation.

### 9.1. Disentanglement Learning-Based Methods

Disentanglement learning is a subpart of representation learning. It aims to give models an optimized representation for a given task. This idea was first proposed by Rifai et al. in 2012 [98] in expression recognition. This idea was firstly applied in UI2IT by Gonzalez-Garcia et al. in 2018 [37], who made the choice to separate attributes into three different spaces—namely, the shared part, which stores the attributes shared across all images, and two exclusive parts that represent the specific attributes inside domains.

Later, Huang et al. [45]. and Lee et al. [99] proposed to separate an image into two spaces, a content space and an attribute space. Another type of disentanglement was proposed by [8,100], who considered the important features as content and the remaining ones as style features.

Recently, Li et al. [101] stated that previous methods are deterministic. They also stated that StarGANv2 [54] makes unnecessary manipulations during the generation (identity transformation or background transformation). They also stipulated that the previous methods do not provide fine-grained control over generation. To alleviate this, they proposed a hierarchical style disentanglement to organize labels as a hierarchical tree structure. For example, the label “glasses” could be disentangled as “sunglasses” and “myopic glasses”. This method proposes more controllable generations and scalability.

Ren et al. [100] began their paper by stating that most content-style disentanglement methods depend on supervision to guide the disentanglement. They followed the work of Gabbay et al. [102] and proposed an unsupervised content-style disentanglement module (C-S DisMo), which tries to isolate the most important features for the reconstruction from less important features. The former are called “content features”, and the latter, “style features”. Another work that tried to separate the content from the style features is that of Liu et al. [8], who separated the content from the style by using a common task called the “domain-invariant high-level vision” task—face segmentation in this case. The most important features for the segmentation are also called content features, and the remaining ones are called style features.

In the same strategy, Kim et al. [22] proposed to give more control over generation. To alleviate that, they proposed a style-aware discriminator that encodes style and sorts real images from generated ones.

In the same vein, Baek et al. [87] stated that recent works rely on guidance available in the data or a paired dataset which constrains the data acquisition. To alleviate that, they introduced a new truly unsupervised image translation (TUNIT) as an UI2IT method that uses a guidance network and two-branch classifier that predicts either the pseudo-label or the style vector.

### 9.2. Contrastive Learning-Based Methods

**Figure 6:**
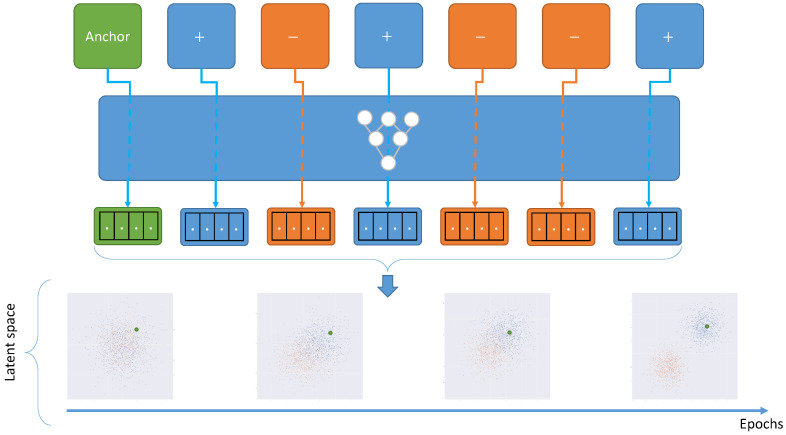
General case of a contrastive learning method. Using anchor and data augmentation methods, the model can learn useful representations.

Contrastive learning is a sub-field of self-supervised learning which consists of making more efficient use of the data. In this case, contrastive learning aims to make a representation with better class separation by adding an objective that constrains the model to add contrast to the learned representation. A visualization example can be found in Figure 6. More deeply, it consists of two pairs, the positive pair which is composed of samples from the same class and the negative pair composed of samples from a different class. The objective function constrains the model to make similar representations for samples that behave in the same class and very different representations for samples in different classes.

To alleviate these problems, contrastive learning-based methods have been proposed to optimize the learned representation, especially with noise contrastive estimation (NCE) [103]. Hereafter in 2020, Park et al. [86] stated that image-to-image translation is a disentanglement learning problem because the goal is to transfer content (which does not change) to a specific style (which has to change). They introduced a contrastive learning technique to UI2IT by using the infoNCE loss to maximize mutual information between patches. Since then, the research community became more inspired by this contrastive learning idea.

One year later, in 2021, Han et al. [104] and Zheng et al. [7] further exploited this idea. The former’s method uses dual contrastive learning using the generator’s encoder and projection layers with a revisited PatchNCE loss to constrain generators during the translation. The latter’s method exploits contrastive learning to make the generator structurally aware using their proposed self-similarity to provide strong supervision on the structure.

Hu et al. [105] found that the patches are sampled randomly, which is not optimal. Given this problem, they proposed to optimize the selection of patches location with a query-selected attention module. Jung et al. [106] mentioned that recent methods did not take semantics into account and proposed semantic relation consistency (SRC) to force the model to keep spatial semantics during the translation and negative mining to further improve the model’s performance by avoiding easy image patches.

Another work that is used in the contrastive learning world is TUNNIT by Baek et al. [87], where a contrastive learning scheme is used in the guiding network. This contrastive learning loss helps the guiding network to extract the style codes.

## 10. Method Comparison

We compare the discussed state-of-the-art current papers by collecting and summarizing their results on different datasets and using different metrics.

**Figure 7:**
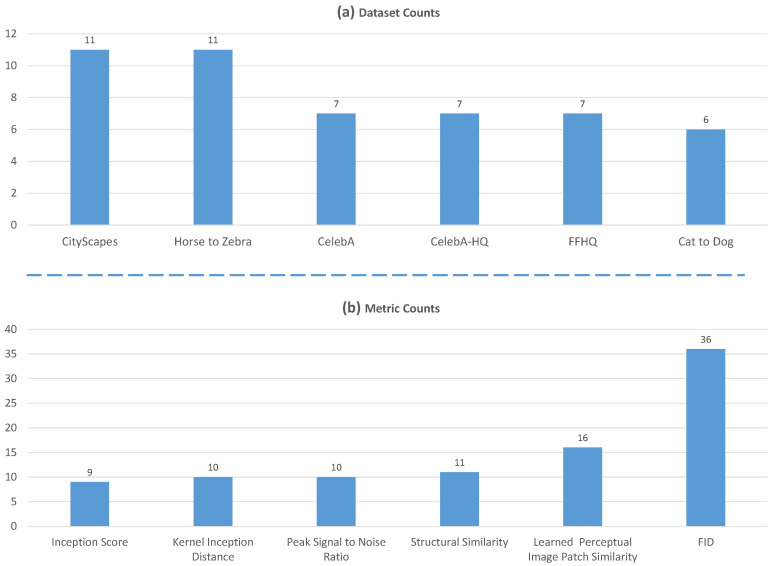
Dataset usage of papers in this review. (**a**) The use of datasets in the papers of this review; (**b**) the metric usage of the reviewed papers.

Figure 7 shows the six most used evaluation datasets in these papers. Cityscapes and Horse to Zebra are the current mainstream datasets for I2I Translation. For face translation methods, CelebA, celebA-HQ and Flickr Face HQ remain the best options for model evaluation.

As Figure 7b shows, LPIPS, SSIM, PSNR and FID are the most used metrics in the reviewed papers, shortly followed by mean intersection over union (mIoU) to check the semantic consistency of recent methods.

In Table 1, we can see the predominance of the FID, as it was nearly used by all studies. This metric can give us an approximation of the network generation quality. According to this metric, LSeSim [7] seems to be the best model in terms of generation quality. However, this statement should be taken with a grain of salt because the authors did not evaluate their method using the SWD. QS-Attn had the best SWD.

In Table 2, we can see that the CitySpaces dataset is useful for checking the semantic translation capabilities of methods. It can benefit from precise segmentation metrics such as pixel accuracy and class accuracy. TSIT obtained the best class accuracy.

In Table 3, we can see that little attention has been given to time or space complexity metrics. This table shows that NICE-GAN is the fastest model.

Since we made a general comparison between papers, we now present a deeper comparison between papers classified in the same category:

Section 5 groups papers that brought progress to the I2I translation field through a change in architecture. These works tried to make faster translations [53,55] and higher resolution translations [59]. Table 4 shows that, on one hand, these methods use mostly least-square adversarial [107] loss and the PatchGAN discriminator [1]. On the another hand, cycle consistency is not used by the majority of methods. As an example, SpatchGAN [59] uses weak cycle loss and Pixel2Style2Pixel [56] uses perceptual loss and regularization.

Section 6 presents more challenging translations that involve large shape modifications. As Table 5 shows that methods in this category do not use the cycle consistency because this cycle consistency imposes a lot of constraints during the training. For example, deformation-aware GAN [64] uses a shape and appearance constraint, TSIT [67] uses perceptual loss and feature matching loss and RSMT [28] proposed a specific type of loss for satellite-to-maps translation, the map consistency loss.

Section 7 groups works that reduce large dataset dependencies and hence increase the usefulness of the data. Table 6 shows the methods extracted from the papers. It shows that these methods do not use cycle consistency. FUNIT [83] and COCO-FUNIT [84] use feature matching loss. ReMiX uses the adversarial loss for translation and another loss to force the generator to preserve the content of the original image.

In Section 8, we can read about works that performed more precise translations on face datasets. From Table 7, we can firstly see that these approaches have a wide variety of adversarial losses. For example, HiFaFace [10] uses high-fidelity domain adversarial loss, and Nederhood et al. [19] used the hinge version of the normal adversarial loss. Furthermore, the methods of this category use a wide range of discriminators. FacialGAN, inspired by StarGAN v2, uses a multitask discriminator. HifaFace [10] proposed a high-frequency discriminator. Nederhood et al. [19] uses the multi-resolution patch-based discriminator from SPADE [108]. In contrast to other categories, these studies mostly used the cycle consistency, except for that of Huang et al. [94], which involved latent cycle consistency. Regarding the used adversarial losses, we can notice that HifaFace proposed to use a new adversarial loss for high-fidelity face editing.

Section 9 groups methods that guide models to the wanted translation. For example, CoMoGAN [97] uses Disentangled Residual Blocks between the encoder and the decoder to guide the generator’s translations. As Table 8 shows, the cycle consistency is not used in these works. CUT prefers to maximize the information between patches and MagGAN [92] using Mask guided reconstruction loss and an attribute classification loss. In the adversarial point of view, these methods use the original adversarial loss [86], the hinge version of the adversarial loss [70,97], least-square adversarial loss [107] or the WGAN-GP adversarial loss [109]. We can also notice that the majority of the proposed methods are multi-modal.

Section 9.1 exhibits a disentanglement mechanism to provide I2I translation models with a better internal representation. Table 9 shows that, due to the disentanglement properties, the cycle consistency is not used in works in this part. Lee et al. [95] used a proposed cross-domain cycle consistency, Liu et al. [8] used a perceptual loss and their proposed domain invariant high-level vision task, and the TUNIT [87] generator is constrained by the style extractor and keeps the domain-invariant feature thanks to the multi-task discriminator outputs. If we focus on losses, we can see the original adversarial loss still has a prominent place over other losses.

Section 9.2 groups studies that used contrastive learning to train their translation models. As Table 10 shows, these methods do not use the cycle consistency. Han et al. [20] followed CUT and chose to maximize the mutual information between patches for the translation and constrained the network with similarity loss to preserve target domain features. Zheng et al. [7] proposed to replace the cycle consistency loss with their proposed Learned self-similarity loss to constrain the network not only to translate, but also to preserve, the structure during the translation. In the adversarial point of view, these works are using the normal adversarial loss.

## 11. Current ChallengesDiscussion

In recent years, the research community has made huge improvements by increasing image resolution, making more challenging translations with the release of Front Face to Profile Face [20], by releasing more lightweight and faster models and developing performance metrics such as sec/iter or the memory consumption. However, such metrics will irrevocably decrease with the advancement of technology. Moreover, the variety of computing stations in the research centers makes this comparison even more difficult. A solution could be to re-run the evaluation for the time of inferences or to find a metric that can take into account the different technologies used and unify time and memory consumption measures.

Moreover, actual deep translation models are constituted by a multitude of parameters and convolution operations. These two characteristics are not compatible with portable devices such as smartphones. Even though ASAPNET [53] came with a lightweight and fast architecture, this proposed generator architecture has not been tested in unsupervised settings.

In addition, even unsupervised models need a big dataset to be correctly trained. Although these datasets can be easily generated or completed by web crawling data [94], we cannot make a dataset for every world case. Whereas translation models that deal with small datasets show good results, such as TUIGAN [68], generation contains artifacts, and the combination of all these models can be computationally intensive.

In Section 8, we saw models that can be guided by additional inputs to match the user’s needs, as is the case for FacialGAN [90]. However, this paper points out a problem that their method does not give full control over generations. This is explained because the discriminator forces the generator to generate realistic faces and hence cannot generate faces with no nose, for example.

One another problem came from the Google Maps dtaset. This dataset can be easily made by scrapping the Google Maps website. However, this causes a consistency problem during the evaluation because the map layout depends on the region where maps are extracted. For example, RSMT [28] shows that a model trained on the Beijing maps does not perform well when tested on Los Angeles because of the structural difference between cities. A good contribution to the satellite-to-map translation field could be the release of a more complete Google Maps dataset with multiple cities in several regions (cities, villages, mountainous landscapes, cities near the sea, highly populated cities and lightly populated cities).

FID is the most used metric in the I2I translation field. This metric is a way to benchmark a given generation model on the state-of-the-art. However, Kynkäänniemi et al. [110] wrote that the FID can be improved without making any improvements by generating a large set of images and selecting images that fit the fringe feature of the real data. Another work from Gu et al. [49] stated that, since FID is based on a model trained on ImageNET, it can be the prey of outliers.

## 12. Conclusions

In this review, we dove into the world of image-to-image translation. Since 2017, this field of research has known many changes and is able to perform translations of higher quality and diversity. Some work also proposed translation models working with small training datasets, and others proposed models that progressively translate an image from one domain to another. Lastly, some work paid attention to geometrical changes of challenging translation. Using a particular splitting to organize papers by the employed method, this review is able to guide quickly the reader to the most useful methods, depending on the reader’s interest. We also noted several drawbacks of the current method and hope this review will contribute to this research field.

## Figures and Tables

**Table 1:** Aggregation of reviewed paper results on the “horse ↔ zebra” dataset. Results taken from papers. Numbers in bold are the results of the best performing models in a given metric. “-” indicates that no results have been found. Results taken from articles.

	Horse ↔ Zebra			
Method	FID ↓	KIDx100 ↓	SIFID ↓	PD ↓
CycleGAN [6]	77.2	4.81	77.2	**3.24**
FastCUT (variant of CUT) [86]	73.4	-	-	-
CUT [86]	45.5	-	-	-
DCLGAN [104]	43.2	-	-	-
SIMDCL [104]	47.1	-	-	-
FSeSim [7]	40.4	-	-	-
LSeSim [7]	38	-	-	-
QS-Attn [105]	42.3	-	-	-
Jung et al. [106]	**34.4**	-	-	-
Nice-GAN [55]	65.9	2.09	-	-
MSPC [20]	61.2	-	-	-
TuiGAN [68]	-	-	**1.03**	6.16
IrwGAN [89]	79.4	**1.83**	-	-
AttentionGAN [51]	68.55	2.03	-	-

**Table 2:** CityScapes results from reviewed papers. Bold numbers are the results of the best performing models in a given metric. Results are taken from papers and “-” indicates that no results have been found. Results taken from articles.

	Photo to Label				
Method	PixAcc ↑	classAcc ↑	IoU ↑	mAP ↑	FID ↓
CycleGAN [6]	55.90	25.40	-	20.40	76.30
MUNIT [45]	-	-	38.30	-	-
Lee et al. [99]	52.70	18.10	13.00	-	-
SCS-UIT[8]	74.00	29.60	-	22.60	-
TUNIT [87]	-	-	35.18	-	-
FastCUT (variant of CUT) [86]	59.90	24.30	-	19.10	68.80
CUT [86]	68.80	30.70	-	24.70	56.40
DCLGAN [104]	74.00	22.00	17.00	-	49.40
SIMDCL [104]	-	-	-	-	51.30
FSeSim [7]	69.40	-	-	-	53.60
LSeSim [7]	73.20	-	-	-	49.70
QS-Attn [105]	**81.40**	32.60	-	27.90	50.20
Jung et al. [106]	73.50	35.60	-	**73.50**	**46.40**
One-to-One CG [57]	52.70	18.10	13.00	-	-
MSPC [20]	74.00	30.00	-	22.60	-
TSIT [67]	65.90	**94.40**	-	-	59.20
ReMix [67]	-	82.70	**70.30**	-	50.10
CoMoGAN [97]	-	-	43.00	-	-

**Table 3:** Performance aggregation of reviewed papers. Bold numbers are the results of the best performing models in a given metric. Results taken from papers and “-” indicates that no results have been found. Results taken from articles.

Methods	Sec/iter ↓	Mem (GB) ↓	Total Net Params (M) ↓
CycleGAN [6]	0.40	4.81	-
StarGANv2 [54]	0.68	-	85.30
HiSD [101]	-	-	-
Style-aware Discriminator [22]	0.38	-	**56.50**
FastCUT (variant of CUT) [86]	0.15	2.25	-
CUT [86]	0.24	3.33	-
DCLGAN [104]	0.41	-	-
SIMDCL [104]	0.47	-	-
FSeSim [7]	-	2.65	-
LSeSim [7]	-	2.92	-
Nice-GAN [55]	**0.15**	**2.25**	-
One-to-One CG [57]	0.24	3.33	-

**Table 4:** Comparison between proposed methods in Architecture Change. In this category (Section 5), we compare papers based on if they use the cycle consistency and the type of adversarial loss. “V” and “X” means that the method use the technique in the columns is “used” and “not used” respectively. “-” denotes that authors did not mention this information.

Methods	Cycle Consistency	Adversarial Loss
NICEGAN [55]	V	Least-square
SpatchGAN [59]	X	Least-square
One-to-One CG [57]	V	Least-square and normal
Richardson et al. [56]	X	-
ASAPNET [53]	X	Hinge-Loss

**Table 5:** Comparison between papers in the Complex Translation category. For this category (Section 6), the comparison was performed based on the cycle consistency utilization and the type of adversarial loss involved. “V” and “X” means that the method use the technique in the columns is “used” and “not used” respectively. “-” indicates that the corresponding information was not found.

Complex Translation	Cycle Consistency	Adversarial Loss
Li et al. [64]	X	Original
ACLGAN [6]	X	Original
MSPC [20]	X	Original
TSIT [67]	X	Hinge
TuiGAN [68]	V	WGAN-GP
CoCosNet V2 [69]	X	Hinge
MapGen-GAN [27]	Cycle and Geometric	Original
RSMT [28]	Map Consistency	Original

**Table 6:** Comparison between papers in the Data Issue category (Section 7). The comparison is based on the cycle consistency and the adversarial loss used. “V” and “X” means that the method use the technique in the columns is “used” and “not used” respectively. “-” indicates that the corresponding information was not found.

Methods	Cycle Consistency	Adversarial Loss
FUNIT [83]	X	Hinge
COCO-FUNIT [84]	X	Hinge
ReMiX [85]	X	Hinge
TransferI2I [26]	X	Original

**Table 7:** Comparison between papers in the Attribute Editing category (Section 8). For this category, the comparison is based on whether a given method uses the cycle consistency, adversarial loss and the discriminator architecture. “V” and “X” means that the method use the technique in the columns is “used” and “not used” respectively. “-” indicates that the corresponding information has not been found.

Attribute Editing	Cycle Consistency	Adversarial Loss	Discriminator Type
FacialGAN [90]	V	Original	Multi-task Discriminator
SART [91]	X	Original	PatchGAN
HifaFace [10]	V	High-frequency Domain	High-frequency Domain Discriminator
Nederhood et al. [19]	X	Hinge	SPADE Discriminator [108]
BridgeGAN [94]	Latent Cycle Consistency	WGAN-GP	Original

**Table 8:** Comparison between papers in the Guidance category (Section 9). This comparison is based on the use of the cycle consistency and the type of adversarial loss used. “V” and “X” means that the method use the technique in the columns is “used” and “not used” respectively. “-” indicates that the corresponding information has not been found.

Guidance	Cycle Consistency	Adversarial Loss
CUT [86]	X	Original
CocosNet [70]	X	Hinge
CoCosNet V2 [69]	X	Hinge
CoMoGAN [97]	X	Least-square
MagGAN [92]	Mask-aware reconstruction error	WGAN loss
DUNIT [95]	V	Content adv loss and Style ad loss

**Table 9:** Comparison between papers in the part disentanglement learning category (Section 9.1). In this table, we made the comparison based on if the cycle consistency is used and the type of adversarial loss used. “V” and “X” means that the method use the technique in the columns is “used” and “not used” respectively. “-” indicates that the corresponding information has not been found.

Disentanglement Learning	Cycle Consistency	Adversarial Loss
DRIT [99]	Cross Cycle Consistency	content adversarial Loss
SCSUIT [8]	X	Least-square
Ren et al. [100]	X	-
HiSD [101]	X	Original
A Style-aware Discriminator [22]	X	Original
TUNIT [87]	X	Original

**Table 10:** Comparison between papers in the Contrastive Learning category (Section 9.2). The comparison is on the use of the cycle consistency and the type of adversarial loss. “V” and “X” means that the method use the technique in the columns is “used” and “not used” respectively. “-” indicates that the corresponding information has not been found.

Contrastive Learning	Cycle Consistency	Adversarial Loss
CUT [86]	X	Original
DCLGAN [104]	X	Original
F/LSeSim [7]	X	Original
QS-Attn [105]	X	Original
Jung et al. [106]	X	-

## Data Availability

Data of the corresponding results can be found in corresponding papers.
